# Comparison of rivaroxaban and parnaparin for preventing venous thromboembolism after lumbar spine surgery

**DOI:** 10.1186/s13018-015-0223-7

**Published:** 2015-05-23

**Authors:** Wei Du, Chunhong Zhao, Jingjie Wang, Jianqing Liu, Binghua Shen, Yanping Zheng

**Affiliations:** Department of Spine Surgery, Yantaishan Hospital, Yantai, 264000 China; Department of Orthopedic Surgery, Qilu Hospital of Shandong University, No. 107 Wenhuaxi Road, Jinan, 250012 China; Department of Hematology, Yantaishan Hospital, Yantai, 264000 China

**Keywords:** Rivaroxaban, Parnaparin, Lumbar spine surgery, Venous thromboembolism

## Abstract

**Background:**

The aim of this study was to evaluate the effectiveness and safety of rivaroxaban for preventing venous thromboembolism (VTE) after lumbar spine surgery.

**Methods:**

In this randomized, controlled study, 665 patients who underwent lumbar surgery were randomly assigned to receive either rivaroxaban or parnaparin. Rivaroxaban and parnaparin were used for preventing postoperative venous thrombosis. The occurrence of postoperative efficacy endpoint events (venous thrombosis) and safety endpoint events (hemorrhage) was compared for each group.

**Results:**

Efficacy endpoint results: in the rivaroxaban group, there were 6 thrombotic events (1.7 %), 2 cases with severe VTE (0.6 %), and 3 cases with symptomatic VTE (0.9 %). In the parnaparin group, there were 10 thrombotic events (3.1 %), 4 cases with severe VTE (1.2 %), and 6 cases with symptomatic VTE (1.9 %). Safety endpoint results: in the rivaroxaban group, there were 21 cases with bleeding events (6.2 %), 2 cases with severe bleeding (0.6 %), and 19 cases with non-severe bleeding (5.6 %). In the parnaparin group, there were 21 bleeding events (6.2 %), 1 case with severe bleeding (0.3 %), and 16 cases with non-severe bleeding (4.9 %). The incidences of thromboembolic events, including severe and symptomatic VTE, were not significantly different between the two groups (*P* > 0.05). Bleeding event rates, including severe and non-severe bleeding, were also not significantly different.

**Conclusions:**

Rivaroxaban proved to be equally effective as parnaparin for anticoagulation therapy, with both drugs exhibiting a similar prevention effect against postoperative VTE after lumbar spine surgery, without increasing the risk of postoperative bleeding.

## Introduction

Deep venous thrombosis (DVT) is one of the more common and dangerous complications of spinal surgery, as it can result in a fatal pulmonary embolism (PE) [1, 2]. The American College of Chest Physicians (ACCP) [2] and the National Institute for Health and Care Excellence (NICE) [3] recommend the use of low-molecular-weight heparin and low-dose common heparin for preventing venous thromboembolism (VTE) among patients who underwent selective surgery and those who suffer from spinal cord injury, and thus are at risk for VTE. Glotzbecker [4] reported that a questionnaire assessing anticoagulant choice among 94 spinal surgeons surveyed revealed that 58 % of surgeons prefer low-molecular-weight heparin as the first choice. Low-molecular-weight heparin is considered the gold standard anticoagulant therapy after spinal surgery because it exerts minimal effects on platelet function and thus has an insignificant influence on the early stages of hemostasis and does not increase the bleeding risk. However, one disadvantage of low-molecular-weight heparin is that it requires subcutaneous absorption. Many patients cannot tolerate the complications caused by long-term subcutaneous injections. Rivaroxaban, a novel oral anticoagulant, has been clinically used following orthopedic surgery, particularly for preventing VTE after replacement surgery of the hip and knee. Although rivaroxaban has been proven to be safe and effective, no report has been published that describes the efficacy and safety of its anticoagulation ability after spinal surgery. The objective of this study is to evaluate the effectiveness and safety of rivaroxaban for preventing VTE after lumbar spine surgery, compared with parnaparin.

## Materials and methods

### Clinical data

The sample size was based on a power analysis with the values α = 0.05, β = 0.1, power = 0.9, and an effect size *P* = 0.8 for parnaparin and *P* = 0.9 for rivaroxaban. These parameters required a sample size of *Z* = 131 for the two groups. Including a withdrawal rate of 20 % suggests that 158 cases should be included.

This clinical trial was a prospective randomized controlled trial that included 978 patients who underwent lumbar surgery between August 2009 and December 2012. According to the high-risk factors of spinal surgery presented in the 7th ACCP vein thrombosis prevention guidelines [2], all cases selected had one of the following high-risk factors for VTE in spinal surgery: 1) age ≥60 years; 2) body mass index (BMI) ≥30 kg/m^2^; 3) thrombophilia, including conditions such as hypertension, diabetes, hyperlipidemia, cancer, and others; 4) previous history of venous thrombosis; 5) anterior or combined anterior–posterior procedure; and 6) spinal trauma or spinal cord damage. Meanwhile, case exclusion criteria included 1) oral anticoagulant therapy 3 months prior to the operation; 2) vein thrombosis on preoperative B-ultrasound; 3) preoperative urinalysis positive for red blood cells, fecal occult blood, skin purpura, or hematoma; 4) active bleeding or high risk of bleeding; and 5) contraindication towards rivaroxaban and parnaparin or patients whose parnaparin dose needed to be adjusted. A total of 665 cases met these criteria and were selected for the experiment. This study was conducted in accordance with the declaration of Helsinki. This study was conducted with approval from the Ethics Committee of Qilu Hospital of Shandong University. Written informed consent was obtained from all participants.

### Grouping

Two groups of envelopes were pre-prepared and labeled with: group A: rivaroxaban and group B: parnaparin. Patients randomly selected one envelope upon admission. Therefore, patients were divided into two groups: the rivaroxaban group (341 cases) and the parnaparin group (324 cases).

### Prevention

Patients in group A began daily oral treatment with 10 mg rivaroxaban (Bayer Schering Pharma AG, Leverkusen, D-51368, Germany) 6 to 8 h after surgery, and the treatment continued until the 14th day, when the patients could fully ambulate. Patients in group B received subcutaneous injections of 40 mg parnaparin (Sanofi-Aventis, France 1–13, Boulevard Romain Rolland 75014 Paris, France) 6 to 8 h after surgery and once per day until the 14th day, when they could fully ambulate.

The study team was composed of researchers from the departments of hematology, anesthesiology, spine surgery, cardiology, pneumology, and ultrasound, along with clinical pharmacists, who participated in the treatment of complications.

### Observation indicators

Patients were monitored every day for symptoms and signs of DVT or PE. The symptoms of DVT included leg swelling, lower extremity pain, and Homans sign. If a symptom or sign was observed, Doppler ultrasound was performed immediately on the bilateral veins of the lower extremities. Symptoms that suggested PE included sudden-onset dyspnea, retrosternal pain, hemoptysis, cyanosis, syncope, profuse sweating, cold limbs, and convulsions. If these were observed, electrocardiography was performed immediately to eliminate other possible conditions, such as myocardial infarction. Spiral computed tomography (CT) was conducted as soon as possible to determine pulmonary angiography. Post discharge, patients were told to inform their caregiver if they experienced any signs and symptoms of DVT or PE. If present, the ultrasound procedure was immediately performed. Asymptomatic patients also underwent Doppler ultrasound on the bilateral deep veins of the lower extremities on the 2nd, 7th, and 14th days of treatment and on the 4th week after the treatment ended. DVT in any area (proximal and distal), non-fatal PE, and the number of patient deaths were recorded.

### Efficacy endpoint indicator

The primary efficacy endpoint was a combined endpoint comprising all DVT, non-fatal PE, and mortality from all causes during the treatment period (until the 14th day). The secondary efficacy endpoint was a combined endpoint composed of severe VTE, i.e., proximal DVT, non-fatal PE, and VTE-induced death during the treatment period (until the 14th day). Other efficacy endpoints included the occurrence of symptomatic VTE during treatment and follow-up and deaths during the follow-up period.

### Safety endpoint indicator

The primary safety endpoint was major bleeding events during the treatment. Severe bleeding was defined as fatal bleeding, bleeding in inflow critical organs (such as the posterior peritoneum, intracranium, intraocular, and intraspinal canal), bleeding-induced reoperation, or clinically significant bleeding outside the surgical site with a decrease of ≥20 g/L in hemoglobin level (with the level from the first postoperative day as the reference value), or the need to transfuse ≥2 units of whole blood or packed red blood cells. The other safety endpoints included all non-serious bleeding (i.e., all events that were not evaluated as severe bleeding, such as skin bruising, gastrointestinal bleeding, fecal occult blood, and urine erythrocytes) during the treatment and bleeding wound complications (a composite indicator of wound hematoma and surgical site bleeding).

### Statistical analysis

SPSS 13.0.1 software was used for statistical analysis. The *χ*^2^ test was employed for comparing the incidence rates of thromboembolic and bleeding events between the two groups. If the number of cases was ≥5, then the *χ*^2^ test was used. If the number of cases was <5, then the corrected *χ*^2^ test was used. If the number of cases was ≤1, then Fisher’s exact test was used. *P* < 0.05 was considered statistically significant.

## Results

### Efficacy endpoint results

In the rivaroxaban group, six thrombotic events occurred (1.7 % of cases). In one case, the patient exhibited femoral vein thrombosis, floating blood clots, and a swollen leg. In another case, the patient demonstrated external iliac vein thrombosis, a swollen leg, difficulty breathing, and a progressive decline in arterial oxygen pressure. Pulmonary artery CT angiography confirmed left upper lobe artery embolization. In another case, the patient had an anterior tibial vein embolism without clinical symptoms. Two cases presented posterior tibial vein thrombosis. In one of these cases, the patient experienced leg swelling and discomfort; in the second case, the patient suffered from peroneal vein thrombosis without clinical symptoms.

In the parnaparin group, 10 thrombotic events occurred (3.1 % of cases). In one case, the patient suffered from external iliac vein and femoral vein thrombosis and suddenly lost consciousness on the 7th day. Lower-extremity B-ultrasound revealed left external iliac vein and femoral vein thrombosis. Heart B-ultrasound revealed pulmonary hypertension, which led to the diagnosis of PE as the cause of death. Femoral vein thrombosis occurred in three cases. In one case, the patient had a swollen leg and difficulty breathing, with a progressive decline in arterial oxygen pressure. This case was considered a non-fatal PE. The patients in the other two cases experienced lower limb swelling and pain. Four cases had peroneal vein thrombosis without clinical symptoms. One case exhibited posterior tibial vein thrombosis with leg pain. During the follow-up period, one case of femoral vein thrombosis with lower limb swelling occurred.

No significant difference was found in the incidence rates of thromboembolic events between the two groups (*P* > 0.05). The incidence rates of severe and symptomatic VTE also exhibited no significant difference (*P* > 0.05, Table 1).

**Table 1:** Occurrence rate of thrombus (efficacy endpoint)

Efficacy endpoint	Rivaroxaban group (case number/total number)	Parnaparin group (case number/total number)	*P* value
Primary efficacy endpoint	6/341 (1.7 %)	10/324 (3.1 %)	>0.25
Death	0/341 (0.0 %)	1/324 (0.3 %)	
Non-fatal pulmonary embolism	1/341 (0.3 %)	1/324 (0.3 %)	
DVT	6/341 (1.7 %)	8/324 (2.5 %)	
Proximal end	2/341 (0.6 %)	3/324 (0.9 %)	
Distal end only	4/341 (1.2 %)	5/324 (1.5 %)	
Severe VTE^a^	2/341 (0.6 %)	4/324 (1.2 %)	>0.25^*^
Symptomatic VTE^b^	3/341 (0.9 %)	6/324 (1.9 %)	>0.25^*^
During the treatment	3/341 (0.9 %)	5/324 (1.5 %)	
During the follow-up	0/277 (0.0 %)	1/268 (0.4 %)	
Death during the follow-up	0/277 (0.0 %)	0/268 (0.0 %)	

^*^Used the corrected *χ* ^2^ test

^a^Severe VTE was the combined turnover, composing of proximal DVT formation, nonfatal pulmonary embolism, and VTE-induced death

^b^Symptomatic VTE included any symptomatic DVT formation (proximal or distal) and nonfatal/fatal symptomatic pulmonary embolism

There were 298 patients who were older than 60 years of age in this clinical trial, and among these, 12 patients (4.0 %) experienced DVT. There were 304 patients with a BMI ≥30 kg/m^2^, among whom 9 (3.0 %) experienced DVT. There were 201 patients who had thrombophilia; of these, 55 cases had diabetes, 2 of whom (3.6 %) experienced DVT; 65 cases had hypertension, 1 of whom (1.5 %) experienced DVT; 20 cases had hyperlipidemia, 1 of whom (5.0 %) had DVT; 32 patients had a combination of 2 of these conditions (diabetes, hypertension, and hyperlipidemia) 2 (6.3 %) of whom also had DVT; 29 cases had spinal tumors, 3 of whom (10.3 %) experienced DVT; and 7 patients had a previous history of venous thrombosis, 1 of whom (14.3 %) experienced DVT. Anterior or anterior–posterior combined surgery was performed on 16 patients, and 1 of these (6.3 %) experienced DVT. Furthermore, 86 cases had spinal trauma; 55 cases had no nerve injury, 1 of whom (1.8 %) experienced DVT; and 31 cases had nerve injury, 4 of whom (12.6 %) experienced DVT (Table 2).

**Table 2:** Distribution of VTE risk factors in DVT patients

VTE risk factor	DVT cases			Total cases enrolled (665)	Ratio of DVT incidence 2.4 %
	Rivaroxaban group (6)	LWMH group (10)	Sum (16)		
Age ≥60 years old	5	7	12	298	4.0 %
BMI ≥30 kg/m^2^	3	6	9	304	3.0 %
Thrombophilia	4	4	6	201	3.0 %
Diabetics	1	1	2	55	3.6 %
Hypertension	0	1	1	65	1.5 %
Hyperlipidemia	0	0	1	20	5.0 %
Diabetics/hypertension/hyperlipidemia	2	0	2	32	6.3 %
Spinal tumor	1	2	3	29	10.3 %
Previous history of phlebothrombosis	1	0	1	7	14.3 %
Anterior or combined anterior–posterior surgery	0	1	1	16	6.3 %
Spinal trauma	2	3	5	86	5.8 %
Without nerve injury	0	1	1	55	1.8 %
With nerve injury	2	2	4	31	12.9 %

Among the 665 patients, anterior surgery was performed in 12 cases (none experienced DVT); anterior–posterior combination surgery was performed in 4 cases (1 experienced DVT), and in this case vertebral tumor section + intra-fixation was performed. Of the other 649 surgery cases, 15 experienced DVT: 2 cases of discectomy, 3 cases of spinal dilatation decompression, 5 cases of fracture reduction (or decompression) intrafixation, 1 case of transforaminal interbody fusion, 1 case of posterior interbody fusion, 2 cases of vertebral tumor resection intrafixation, and 1 case of a secondary overhauling operation. The secondary overhauling operation included: 5 cases of posterior interbody fusion after the post-disc surgery recurrence, 1 case of a posterior overhauling operation and DVT occurrence (this patient did not heal 6 months after the fracture surgery and exhibited an autonomic nerve fracture), and 1 case of a second focus cleaning because DVT occurred within 1 year of tuberculose focus cleaning interbody intrafixation (Table 3).

**Table 3:** Postoperative DVT of 665 posterior surgery patients distributed according to the surgical methods

Surgical method	Cases (665)	DVT cases		
		Rivaroxaban (6)	Low-molecular weight heparin sodium (10)	Sum (16)
Anterior	12	0	0	0
Fracture reduction decompression intrafixation	6	0	0	0
Vertebral tumor section intrafixation	4	0	0	0
Tuberculose focus cleaning bone graft intrafixation	2	0	0	0
Anterior + posterior	4	0	1	1
Vertebral tumor section intrafixation	3	0	1	0
Tuberculose focus cleaning interbody intrafixation	1	0	0	0
Posterior	649	6	9	15
Vertebral tumor section	260	1	1	2
Spinal dilatation decompression	166	1	2	3
Fracture reduction (or decompression) intrafixation	80	2	3	5
Intervertebral foramen approach vertebral interbody fusion (TLIF)	52	0	1	1
Posterior vertebral interbody fusion (PLIF)	37	1	0	1
Tuberculose focus cleaning interbody intrafixation	8	0	0	0
Vertebral tumor section intrafixation	22	1	1	2
Vertebral tumor section	16	0	0	0
Second overhauling operation	8	0	1	1

### Safety endpoint results

In the rivaroxaban group, 21 cases (6.2 %) experienced bleeding events, with 2 cases of severe bleeding. In one of the severe bleeding cases, a reoperation for epidural hematoma-induced neurological dysfunction was performed on the 4th postoperative day. In the other case, the patient needed a reoperation for an arterial bleeding-induced edge hematoma on the 4th postoperative day. A total of 19 cases of non-serious bleeding were observed. Six of these cases occurred in the subcutaneous large ecchymosis outside the incision site; 7 cases involved incision bleeding (with 3 cases being infected); 5 cases were suspected gastrointestinal bleeding (with positive fecal occult blood); and the urinalysis result of 1 case was positive for red blood cells.

In the parnaparin group, 17 patients (5.2 %) experienced bleeding events. In one case, the patient suffered from severe bleeding that was likely caused by a gastrointestinal bleeding event (e.g., vomiting, bloody diarrhea) and low hemoglobin (70 g/L) level. This patient was transfused with four units of red blood cells. Furthermore, 16 cases of non-serious bleeding were observed. Among these, 2 were cases of spontaneous hematoma outside the incision sites, 4 were cases of large-area of ecchymosis, and 4 were cases of bleeding incision (with 2 cases being infected). Four patients were suspected of having alimentary tract hemorrhage, with positive fecal occult blood. The urinalysis results of 2 cases were positive for red blood cells.

No significant difference was found in the bleeding rates of the thromboembolic events between the two groups (*P* > 0.05), with severe bleeding and non-severe bleeding also showing no statistically significant differences (*P* > 0.05, Table 4).

**Table 4:** Occurrence rate of bleeding (safety endpoint)

Bleeding events	Rivaroxaban group	Parnaparin group	*P* value
Any bleeding during the treatment	21 (6.2 %)	17 (5.2 %)	>0.5
Severe bleeding	2 (0.6 %)	1 (0.3 %)	>0.25^*^
Fatal bleeding	0	0	
Bleeding into important organs	1	0	
Reoccurrence of surgical bleeding	1	0	
Clinically obvious bleeding outside the surgical site	0	1	
Induced hemoglobin decreasing	0	0	
Induced blood transfusion ≥2 units	0	0	
Non-severe bleeding	19 (5.6 %)	16 (4.9 %)	>0.5
Clinically related non-severe bleeding^a^	6	6	
Bleeding incision complications^b^	7	4	
Other non-severe bleeding	6	6	

^*^Used Fisher’s exact test

^a^Clinically related non-severe bleeding included multi-sites bleeding, spontaneous hematoma and big incision hematoma

^b^Bleeding incision complications was the combined indicator of big incision bleeding and surgical site bleeding

## Discussion

DVT is one of the more common and dangerous complications of spinal surgery, as it may cause fatal PE [1, 2]. Many factors contribute to VTE after spinal surgery, including 1) age ≥60 years; 2) BMI ≥30 kg/m^2^; 3) thrombophilia, including conditions such as hypertension, diabetes, hyperlipidemia, cancer, or others; 4) previous history of venous thrombosis; 5) an anterior or combined anterior–posterior procedure; and 6) spinal trauma or spinal cord damage. There were also some confounding factors related to the operation, such as position (prone position leads to increased abdominal pressure and DVT), anesthesia method (nerve block can cause venous dilatation of the lower extremity, slowed circulation, and DVT), and operation route (anterior lumbar surgery always slows circulation, which carries a high risk of DVT). At the 2009 Annual Meeting of the North American Spine Society, certain postoperative precautions were recommended after spinal surgery to reduce the incidence of thrombotic events [5]. The ACCP [2] and NICE [3] recommended the use of low-molecular-weight heparin and low-dose common heparin for preventing VTE among patients who underwent a selective operation and who suffer from spinal cord injury and thus are at risk of VTE. Low-molecular-weight heparin was chosen as the first-line postoperative anticoagulation drug and the gold standard for spinal surgery [4, 6]. However, low-molecular-weight heparin has the disadvantage of requiring subcutaneous absorption. Several patients cannot tolerate the complications caused by long-term subcutaneous injections. Rivaroxaban is a recently developed oral anticoagulant. The use of rivaroxaban has gradually extended to orthopedic surgery. However, its efficacy for spinal surgery and postoperative safety assessment has rarely been reported. Thus, we evaluated the effectiveness and safety of rivaroxaban for preventing VTE after lumbar spine surgery, compared with parnaparin.

This study found the following efficacy endpoint results: in the rivaroxaban group: 6 cases of thrombotic events (1.7 %), 2 cases of severe VTE (0.6 %), and 3 cases of symptomatic VTE (0.9 %); in the parnaparin group: 10 cases of thrombotic events (3.1 %), 4 cases of severe VTE (1.2 %), and 6 cases of symptomatic VTE (1.9 %). The safety endpoint results are as follows: in the rivaroxaban group: 21 cases of bleeding events (6.2 %), 2 cases of severe bleeding (0.6 %), and 19 cases of non-severe bleeding (5.6 %); in the parnaparin group: 21 cases of bleeding events (6.2 %), 1 case of severe bleeding (0.3 %), and 16 cases of non-severe bleeding (4.9 %). In this study, the incidences of thromboembolic events, i.e., severe and symptomatic VTE, were not significantly different between the two groups (*P* > 0.05). Bleeding event rates, i.e., severe and non-severe bleeding, were also not significantly different. Therefore, rivaroxaban prevented VTE to a similar degree as parnaparin after lumbar spine surgery, without increasing the risk of postoperative bleeding over that of parnaparin.

The pharmacological mechanism of rivaroxaban involves directly antagonizing blood coagulation factor X to achieve its anticoagulation properties. Rivaroxaban exhibits high oral bioavailability. Drug trials show that its anticoagulant response is predictable and thus does not require monitoring. The results of completed phase II and III clinical trials showed that rivaroxaban exhibits excellent DVT prevention after hip or knee replacement surgery. One study that used rivaroxaban as the routine anticoagulation therapy for 3449 cases of hip and knee replacement surgeries showed that rivaroxaban displayed similar anti-thrombotic efficacy to classic heparin but did not increase the risk of bleeding [7]. Given that it is easy to administer, does not require long-term laboratory parameter monitoring, and possesses other unique advantages, the use of rivaroxaban has gradually extended to orthopedic surgery. However, reports describing its efficacy and postoperative safety assessment after spinal surgery are rare.

Early postoperative use of anticoagulant drugs may cause hemorrhagic events, such as increased incision bleeding, formation of a wound hematoma, and intraspinal hematoma, thus increasing the postoperative risk [8, 9]. This is the main reason why anticoagulants are not used by several spine surgeons. Low-molecular-weight heparin is considered the gold standard anticoagulant therapy after spinal surgery because it exerts a minimal effect on platelet function and thus has an insignificant influence on the early stages of hemostasis and does not increase bleeding risk [10]. Several reports, such as those by Hebbeler [11] and Ploumis [12], confirmed that using low-molecular-weight heparin after spinal surgery does not increase the risks of incision bleeding and spinal hematoma formation. Rivaroxaban, as an X factor antagonist, was reported to exhibit rapid metabolism in the plasma in pharmacological experiments. In addition, its anticoagulant response was predictable, and thus, rivaroxaban would not cause a significantly prolonged prothrombin time or activated partial thromboplastin time. The study performed to determine the optimal dosage of rivaroxaban for patients undergoing major orthopedic surgeries found that there was a close correlation between the pharmacokinetics and pharmacodynamics of rivaroxaban, and that 10 mg rivaroxaban (once daily) was a suitable dose in the phase III clinical trial, without the need for dosage adjustment [13, 14].

The results of the completed clinical trial confirmed that the prophylactic dose of rivaroxaban (10 mg oral, once daily) for anticoagulation therapy after hip and knee replacement surgery successfully lowered the risk for DVT and did not increase the risk of bleeding [15, 16].

Spinal epidural hematoma and its neurological sequelae are the most serious complications associated with anticoagulation drugs in perioperative spinal surgery, with a reported incidence ranging from 0.1 to 0.7 % [10, 17, 18]. In the current clinical trial, one case (0.3 %) of epidural hematoma was reported in the rivaroxaban group. However, this complication also occurred in the absence of an anticoagulant. The most prominent reason for spinal epidural hematoma surgery is a coagulation disorder with various causes, such as liver or kidney disease, alcohol abuse, or radiation or chemotherapy, which can lead to spontaneous bleeding [19]. Using logistic regression assessment risk factors, Kou et al. [17] posited that only when a patient had coagulation disorders with multiple steps would he or she be at a high risk for spinal epidural hematoma. The typical symptom of spinal epidural hematoma is progressive neurological deficit, and it is not typical for back pain to be the only symptom. In a study involving 13 reported cases of postoperative spinal epidural hematoma, Gerlach [10] found that newly occurring neurological impairment occurred in approximately 77 % of such cases. Once spinal epidural hematoma is diagnosed, early surgical decompression is the best solution for restoring nerve function. Vandermeulen et al. [20] reported that nerve function could recover fully within 8 h after the onset of symptoms with surgical decompression. Gerlach et al. [10] reported that the postoperative nerve function of 60 % of patients who underwent early surgical decompression could recover completely. The remaining patients who suffered from postoperative bleeding due to spinal cord injury were being treated with anticoagulant drugs. In another study evaluating 476 spinal cord injury patients, bleeding at surgical sites and in the upper digestive tract accounted for 16 and 19 % of bleeding complications, respectively [21]. No incidence of epidural hematoma was reported.

The ACCP recommendations state that prevention is no longer necessary for patients with selective spinal surgery who do not have multiple risk factors [2]. However, the majority of patients who undergo spinal surgery are elderly or have other risk factors. In this experiment, the DVT incidence rates of the patients who had a previous history of phlebothrombosis, a combination of spinal trauma and nerve injury, spinal tumor, or a combination of two conditions out of diabetes, hypertension, and hyperlipidemia, were 14.3, 12.9, 10.3, and 6.3 %, respectively, indicating that these four factors were high-risk factors for DVT after spinal surgery. These patients should be given early postoperative parnaparin or rivaroxaban and intermittent pneumatic compression treatment. The prevention of thrombosis among spinal fracture patients often requires multidisciplinary expertise, including spinal surgery and trauma surgery, from physicians of various disciplines, to complete the treatment. When no obvious endogenous or exogenous bleeding tendency is observed, parnaparin and intermittent pneumatic compression treatments should be applied as soon as possible to prevent VTE. Premature chemoprophylaxis may increase the risk of bleeding complications, such as epidural hematoma. However, the risk/benefit ratio supports early prevention over observation. Therefore, physicians should be aware of the bleeding tendencies of their patients. Once a diagnosis of epidural hematoma is confirmed, early surgical decompression will be more favorable for recovering nerve function. Given the particularity of spinal surgery, once a postoperative spinal hematoma is formed, the risks of secondary damage to the spinal cord and nerves will undoubtedly increase, particularly in the cervical and thoracic spine where the effective volume of the spinal canal is small. The hematoma event will undoubtedly increase the chance of secondary nerve damage in a duplicative manner. Given that no previous safety assessment of the use of rivaroxaban as an anticoagulant therapy after spinal surgery is available, we fully considered the risk of spinal hematoma or bleeding-caused nerve damage during anticoagulation when we designed the clinical trials in this study. Because of this, we likely minimized the risk of secondary neurological damage. Therefore, only patients with lumbar cases were treated with rivaroxaban in this initial safety study. Based on the analysis of anatomical characteristics, the coccygeal nerve root of the lower lumbar nerve exhibits a certain tolerance to external compression due to complications, and the effective volume of the lower lumbar spinal canal was relatively large and had a buffer space. Therefore, the prompt removal of the hematoma would result in a low probability of irreversible neurological damage.

This study showed that, compared with parnaparin, the new oral anticoagulant rivaroxaban was easy to administer and exhibited a reliable anticoagulant effect, which could reduce postoperative thrombosis events in patients at a high risk for VTE. Compared with parnaparin, rivaroxaban did not increase the postoperative bleeding risk during lumbar spine surgery. However, because of differences in spinal anatomy, the spinal cord and cauda equina exhibited different tolerance levels to external compression. Whether rivaroxaban can be extensively used in cervical and thoracic postoperative anticoagulation still requires careful consideration and should be validated using large-scale, multi-center, randomized, and double-blind clinical trials.

## Conclusions

In conclusion, VTE is a complication that can cause fatal damage after spinal surgery (Table 1). This study found four risk factors that presented the highest risk for post-spine surgery VTE: previous history of venous thrombosis, spinal trauma associated with nerve injury, spinal tumors, and the combination of two of three conditions (diabetes, hypertension, or hyperlipidemia) (Table 2). Finally, this study demonstrated that rivaroxaban had a similar efficacy as parnaparin for preventing postoperative VTE after lumbar spine surgery, without increasing the risk of postoperative bleeding (Table 4).
